# NEDD4-1 Regulates Migration and Invasion of Glioma Cells through CNrasGEF Ubiquitination In Vitro

**DOI:** 10.1371/journal.pone.0082789

**Published:** 2013-12-10

**Authors:** Hao Zhang, Wenchen Nie, Xu Zhang, Gentang Zhang, Zhiqiang Li, Huaibing Wu, Qiong Shi, Yong Chen, Zhijun Ding, Xiuping Zhou, Rutong Yu

**Affiliations:** 1 Department of Neurosurgery, Affiliated Hospital of Xuzhou Medical College, Xuzhou, Jiangsu, China; 2 Lab of Neurosurgery, Xuzhou Medical College, Xuzhou, Jiangsu, China; 3 The Graduate School, Xuzhou Medical College, Xuzhou, Jiangsu, China; Ospedale Pediatrico Bambino Gesu', Italy

## Abstract

Neuronal precursor cell-expressed developmentally down-regulated 4-1 (NEDD4-1) plays a great role in tumor cell growth, but its function and mechanism in cell invasive behavior are totally unknown. Here we report that NEDD4-1 regulates migration and invasion of malignant glioma cells via triggering ubiquitination of cyclic nucleotide Ras guanine nucleotide exchange factor (CNrasGEF) using cultured glioma cells. NEDD4-1 overexpression promoted cell migration and invasion, while its downregulation specifically inhibited them. However, NEDD4-1 did not affect the proliferation and apoptosis of glioma cells. NEDD4-1 physically interacted with CNrasGEF and promoted its poly-ubiquitination and degradation. Contrary to the effect of NEDD4-1, CNrasGEF downregulation promoted cell migration and invasion, while its overexpression inhibited them. Importantly, downregulation of CNrasGEF facilitated the effect of NEDD4-1-induced cell migration and invasion. Interestingly, aberrant up-regulated NEDD4-1 showed reverse correlation with CNrasGEF protein level but not with its mRNA level in glioma tissues. Combined with the in vitro results, the result of glioma tissues indicated post-translationally modification effect of NEDD4-1 on CNrasGEF. Our study suggests that NEDD4-1 regulates cell migration and invasion through ubiquitination of CNrasGEF in vitro.

## Introduction

Malignant glioma is the most common and lethal tumor in the central nervous system and is histologically graded as I-IV by World Health Organization (WHO) based on four main features-nuclear atypia, mitosis, microvascular enrichment, and necrosis[1-3]. Grade I and II are low-grade gliomas, while higher-grade gliomas like grade III and IV are malignant and have increased proliferative and invasive capacity [1,4,5]. Despite advances in surgery and adjuvant therapy, the median survival time of patients with malignant glioma has changed little over recent decades [2,3]. A major cause of the failure of conventional treatments is the highly invasive and diffusively inﬁltrative nature of these tumors [6,7]. Therefore, seeking molecular mechanisms of glioma invasiveness is expected to develop effective therapeutic targets for this incurable cancer [4].

Poly-ubiquitination of proteins for degradation has been implicated in cancer development and attracted more and more attention [8-12]. Since E3-ubiquitin ligase, which is responsible for substrate recognition, is also the key regulatory targets in the poly-ubiquitination process, E3-ubiquitin ligase thus becomes the “hot spot” of the study [13,14]. A prominent family of E3-ubiquitin ligase is the neural precursor cell expressed, developmentally down-regulated 4 (NEDD4) family[15-22]. NEDD4-1, a member of NEDD4 family, has a catalytic C-terminal HECT domain, N-terminal C2 and WW domains responsible for substrate recognition [23-25]. By ubiquitinating various substrates, NEDD4-1 regulates many physiological functions, such as cellular proliferation and organism growth, water balance, T cell function [26], the development of neuromuscular junction [27], development of central nervous system and axon guidance [16,28,29], and brain diseases [30,31]. NEDD4-1 positively regulates growth and proliferation of cells especially during embryonic development and NEDD4-1 knockout induces growth retardation and associated perinatal lethality [32].

Recently, lots of studies show that NEDD4-1 is involved in cancer development [17,33,34]. For example, NEDD4-1 is highly expressed in a wide variety of tumors, such as colorectal cancer, bladder cancer, gastric carcinoma [35]. It is reported that NEDD4-1 is identified as the E3-ubiquitin ligase that promotes ubiquitin-mediated phosphatase and tensin homologue (PTEN) degradation, activating PI3K/AKT signaling pathway and promoting cell proliferation [33]. But there is no report about the role of NEDD4-1 in cell invasive behavior, an important feature of gliomas. In this study we investigated the function and mechanism of NEDD4-1 in glioma cell migration and invasion in vitro. We found that NEDD4-1 promoted the glioma cell migration and invasion via triggering cyclic nucleotide Ras guanine nucleotide exchange factors (CNrasGEF) ubiquitination and degradation. 

## Materials and Methods

### Cell culture

The human glioma cell line U251 and U87 were purchased from Shanghai Cell Bank, Type Culture Collection Committee,Chinese Academy of Sciences (CAS). Cells were cultured in Dulbecco’s modified Eagle’s medium and F-12 (DMEM/F-12) (Invitrogen), supplemented with 10% fetal bovine serum (Sijiqing Biological Engineering Materials Co., Ltd.) and grown in a humidified incubator with 5% CO_2_ at 37°C. In some experiments, U251 cells were treated with MG132 at 25 μM final concentrations for 16h.

### Plasmids, antibodies and reagents

The pcDNA3.1-NEDD4-1 (HA-NEDD4-1) was kindly donated by professor Jiang from Memorial Sloan-Kettering Cancer Center in U.S.A [33]. The pGPU6/GFP/Neo-NEDD4-1 was generated by Shanghai GenePharma Co. The pBlueskript-SKII-CNrasGEF was purchased from Kazusa DNA Research Institute in Japan. HA-ubiquitin vector was kindly donated by Dr. Zhenge Luo from Institute of Neuroscience, Shanghai Institutes for biological Sciences, Chinese Academy of Sciences. The following antibodies were purchased-mouse anti-HA(Abmart, Shanghai), rabbit anti-CNrasGEF (residue 975 and 1025 of human PDZ domain) (NOVUS), rabbit anti-NEDD4-1 (H-135, Santa Cruz Biotech). MG132 was obtained from Calbiochem. 

### Transient transfection of plasmids and small interfering RNAs

Transfection of glioma cells with pcDNA3.1-NEDD4-1, pGPU6/GFP/Neo-NEDD4-1 (shNEDD4-1), HA-ubiquitin, pBlueskript-SKII-CNrasGEF and small interfering RNAs targeting human CNrasGEF was performed by using the Lipofectamine™ 2000 transfection reagent (Invitrogen) according to the manufacturer’s instructions. The shRNA sequences (5′-3′) targeting human NEDD4-1 at the nucleotide site 556 was listed below (target sequences was underlined). Sense: 5’-CACCGCAGAACAGGCTGAGGAATTATTCAA GAGATAATTCCTCAGCCTGTTCTGCTTTTG-3’; Antisense: 5’-GATCCAAAAAAGCAGAACAGGCTGAGGAATTATCTCTTGAATAATTCCTCAGCCTGTTCTGC-3’. Three sets of siRNA duplexes (Shanghai GenePharma Co.) targeting human CNrasGEF were listed below.

CNrasGEF-784 (siCNrasGEF-1) 5’-CAGGGACCAUAGUGUUUAATT-3’;
CNrasGEF-2785 (siCNrasGEF-2) 5’-GGGAGAAACUUCCCAAUAATT-3’;
CNrasGEF-4069 (siCNrasGEF-3) 5’-GUGGCUCCCAUGAUAAUAUTT-3’;

All the transfections were performed three times independently.

### RNA extraction and reverse transcription-polymerase chain reaction

Total RNA of glioma samples and cells was extracted using the TRIzol Reagent (Tiangen Biotech CO.). First-strand cDNA was synthesized in a 20 μl reaction volume using the Reverse Transcription Reagents (TaKaRa RNA PCR Kit (AMV) Ver.3.0, TaKaRa Biotechnology, Dalian, China) according to the manufacturer’s instructions. Primers (Sangon Biotech Co.) for NEDD4-1 were as follows: sense: 5’-TCGGTTGGAGAATGTAGC-3’; antisense: 5’-GGGTATAATTGTCCGTAGC-3’ yielding a 341bp product. Primers (Sangon Biotech Co.) for CNrasGEF were: sense: 5’-GAGGCTCTGAGAAGGGATTTGGA-3’; antisense: 5’-ATCTGGAATGGAGTTAGCGACTGG-3’ yielding a 305bp product. And primers for β-actin were: sense:5’-CTGGGACGACATGGAGAAAA-3’; antisense: 5’-AAGGAAGGCTGGAAGAGTGC-3’ yielding a 564bp product as a reference for normalization. Polymerase chain reaction conditions were as follows: 94°C, 3 minutes; 95°C, 30 seconds; 61°C, 30 seconds; 72°C, 35 seconds, and an additional 28 cycles (for NEDD4-1), or 30 cycles (for CNrasGEF), or 22 cycles (for β-actin); 72°C, 7 minutes. 

### Wound-healing assay

Twenty-four hours after transfection, a rectangular lesion on monolayers was created with a pipette tip [36-38]. Cells were washed at least three times with PBS to remove debris and then cultured in serum-free DMEM. At the designated time, five randomly selected fields at the lesion border were acquired under an inverted microscope (Olympus, IX71). The number of migratory cells was measured by Image J software. Data were obtained from three independent assays performed in triplicate.

### Cell invasion assay

Cell invasion assay was performed using a transwell system that incorporated a polycarbonate filter memebrane with a diameter of 6.5 mm and pore size of 8μm (Corning, NY), according to the manufacturer’s protocol[36-38]. To assess invasion, ﬁlters were precoated with 10μg of matrigel (BD Biosciences). A pretreated cell suspension (1 × 10^5^) in serum-free culture media was added to the inserts, and each insert was placed in the lower chamber filled with culture media containing 10% FBS as a chemoattractant. Twenty-four hours after incubation at 37°C, the non-invasive cells were removed from the upper chamber; ﬁlters were ﬁxed with methanol for 15 min and stained with a 0.1% crystal violet solution for 10 min. Five fields of adherent cells in each well were randomly photographed under an inverted microscope and counted. All the experiments were performed three times independently.

### Cell proliferation and apoptosis assay

The effect of NEDD4-1 and shNEDD4-1 on glioma cell proliferation was obtained by detecting the cell viability with the Cell Counting Kit-8 (CCK-8, Beyotime) 24h after transfection according to the manufacturer’s instruction. Cell viability measurements were normalized to those taken at 0 hour in each group. Flow cytometry assay was used to study the effect of NEDD4-1 and shNEDD4-1 on cell apoptosis, which was carried out using the Vybrant Apoptosis Assay Kit #2 (Invitrogen). After being transfected with NEDD4-1 or shNEDD4-1, the cells were harvested and stained with PI and Annexin V according to the manufacturer's protocol. In each sample, 1×10^6^ cells were assayed on a FACSCalibur (Becton-Dickinson) and analyzed by CellQuest Pro software (Becton-Dickinson).

### Immunoprecipitation

Proteins were extracted from U251 cells using the NP-40 buffer (2μg/ml Aprotinin, 10 μM Leupeptin, 1% NP-40, 1μM Pepstatin A, 0.5mM PMSF, 4mM Benzamidine, 1mM DTT) for 30min at 4°C. Protein concentration was detected using the BCA Protein Assay Kit (Thermo scientific) and 500μg lysates were used for immunoprecipitation. Lysates were then incubated with primary antibodies (2μg rabbit anti-NEDD4-1, 2μg rabbit anti-IgG, or mouse anti-HA) and 30μl protein G agrose beads at 4°C overnight. The beads were washed with NP-40 buffer and then centrifuged in 1000g for 2 min at 4°C for 5 times. The beads and antigen banding antibodies complex were boiled and detected by western blotting [39].

### Western blotting

Forty-eight hours after transfection, proteins were extracted from cells using the NP-40 buffer (2μg/ml Aprotinin, 10 μM Leupeptin, 1% NP-40, 1μM Pepstatin A, 0.5mM PMSF, 4mM Benzamidine, 1mM DTT) for 30min at 4°C. Protein concentration was detected using the BCA Protein Assay Kit (Thermo scientific) and equal amount of protein lysates were subjected to 12% sodium dodecyl sulfate-polyacrylamide gel electrophoresis, then transferred to polyvinylidene difluoride membrane of 0.45-μm pore size (Millipore), and probed with primary antibodies (CNrasGEF, NEDD4-1, HA, and β-actin) at 4°C overnight and secondary antibodies at room temperature for 2 hours. Bound antibodies were detected by the Pierce ECL Plus Western Blotting Substrate (Thermo Fisher Scientific Inc.) and exposed to X-ray films. Band densities were quantified by Image-Pro Plus Software (Media Cybernetics, Inc.) and the densitometric results were shown. The relative amount of proteins was determined by normalizing the densitometry value of interest to that of the internal loading control. Western blot experiments were carried out in three biological replicates and average fold changes were reported.

### Glioma and nontumorous human brain tissues

Surgically removed human glioma tissues (n=15) and nontumorous brain tissues (n=8) were obtained frozen from Affiliated Hospital of Xuzhou Medical College (Xuzhou, China). Gliomas were graded by the Pathology Department of Affiliated Hospital of Xuzhou Medical College based on the World Health Organization grading system[40]. As controls, human normal brain tissues (mostly from the cortex) were obtained from patients with decompressive surgery after physical injuries to the brain. These specimens were collected from the patients registered at the above-mentioned hospital and written informed consent was obtained from the patients. This study was approved by the ethics committees of the hospital. The Ethics Committee of the Affiliated Hospital of Xuzhou Medical College consists of the following people: Chairman: Tie Xu; Vice chairman: Yuming Gu; Boards: Junnian Zheng; Zeqiang Ren; Hong Zhu; Yiming Li; Peisheng Jin; Tiejun Cui; Fang Tian; Yuxia Liu; Dongye Li; Xia Shen; Chun Dai; Xiangnong Li; Zhongming Zhang; Jiachun Chen; Longzhen Zhang; Xiuying Pan; Ying Zhou; Dongmei Lv; Guang Zhao.

### Immunohistochemistry

Paraffin-embedded sections were obtained from the pathology files of the Department of Pathology at the Affiliated Hospital of Xuzhou Medical College including 15 astrocytomas of WHO grade II, 9 anaplastic astrocytomas of WHO grade III, 5 glioblastomas of WHO grade IV. Nontumorous brain specimens were acquired from eight patients under-going surgery for internal decompression in cerebral trauma and were reviewed to verify the absence of tumor. Paraffin sections (5µm thick) were baked at 60°C, deparaffinized in xylene and rehydrated in graded ethanol, and then microwaved for antigen retrieval. These sections were treated in 3% hydrogen peroxide for 30mins. Slides were incubated at 4°C overnight with anti-NEDD4-1 antibody (1:200 dilution; Santa Cruz Biotech.) or anti-CNrasGEF antibody (1:200 dilution; Sigma). The bound antibodies were detected by use of the streptavidin-peroxidase kit, which includes biotinylated anti-rabbit IgG (secondary antibody) and peroxidase-labeled streptavidin (Beijing Zhongshan Golden bridge Bio). After the above steps, the slides were counterstained with hematoxylin, dehydrated with ethanol and xylene, and covered with coverslips. Micrographs were taken and the results are presented as the percentage of the glioma cells with positive staining. NEDD4-1 and CNrasGEF staining were categorized on the basis of percentage of positive cells on the slide (0, no expression;+, 1-30%;++, 31-65 %;+++, >65 %). IHC stainings were performed at least twice with similar stained patterns [36].

### Statistical Analysis

One-way ANOVA and Student’s *t*-test were used for data analysis. In all analysis, quantitative data were obtained from at least three independent experiments and expressed as means ± SEM. *P* values less than 0.05 were considered statistically significant (*P<0.05, **P<0.01).

## Results

### Effects of NEDD4-1 on cell migration and invasion

**Figure 1:**
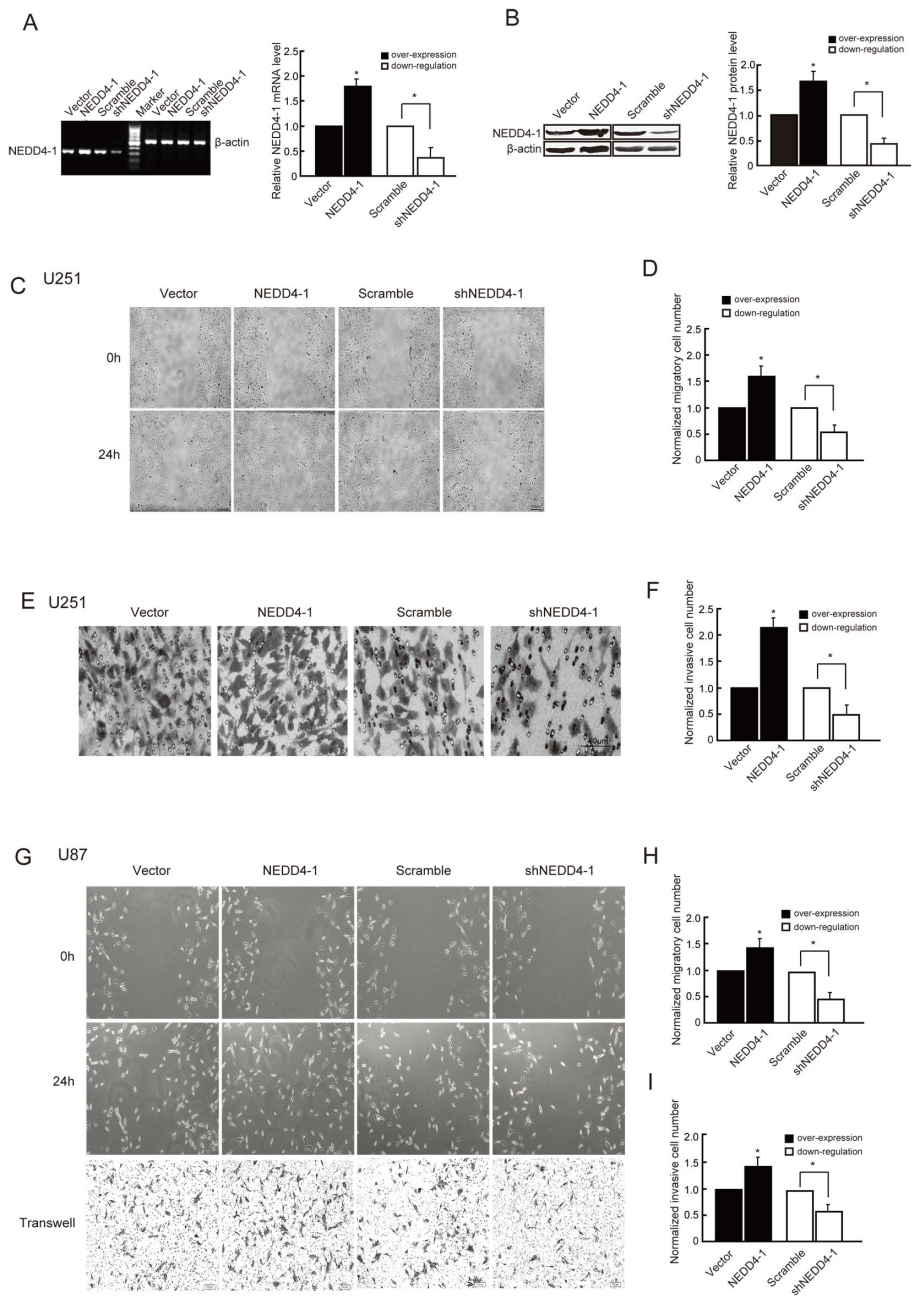
The effect of NEDD4-1 on cell migration and invasion. A) NEDD4-1 overexpressing or downregulating efficacy in U251 glioma cells examined by RT-PCR. Left, Representative RT-PCR analysis. Twenty-four hours after transfection, total RNAs were extracted and underwent RT-PCR analysis. β-actin was used as internal control. Right, Quantitative analysis of relative mRNA levels of NEDD4-1 normalized to those of β-actin. B) NEDD4-1 overexpressing or downregulating efficacy in U251 glioma cells detected by western blotting. Left, Representative image of western blotting. Forty-eight hours after transfection, cells were lysed and protein extraction was underwent western blot analysis using NEDD4-1 antibody. β-actin was used as the loading control. Right, Quantitative analysis of relative protein levels of NEDD4-1 normalized to those of β-actin. C) Wound-healing assay of glioma U251 cells after NEDD4-1 overexpression or downregulation. Representative digital pictures were taken at 0h and 24h. Bar: 100 μm. D) Quantitative analysis of the number of migratory cells. E) Transwell invasion assay of glioma U251 cells after NEDD4-1 overexpression or downregulation. After 48 hours of transfection, cell suspension was added into the matrigel precoated transwell chambers and the cells invaded through the matrigel were stained and photoed. Bar: 50 μm. F) Quantitative analysis of the number of invasive cells. G) Wound-healing (up and middle panel) and transwell invasion assay (bottom panel) of glioma U87 cells after NEDD4-1 overexpression or downregulation. Bar: 100 μm. H & I) Quantitative analysis of the number of migratory (H) or invasive (I) cells. Results are mean ± SEM of three independent experiments in triplicate. *P<0.05.

Firstly, we examined whether NEDD4-1 has a role in cell migration and invasion using gain-of and loss-of function approach in glioma cells. After being transfected with pcDNA3.1-NEDD4-1[33] or pGPU6/GFP/Neo-NEDD4-1 (shNEDD4-1) for 48h, the total RNA of cells was extracted and examined by RT-PCR. As is shown in Figure 1A, compared with the vector and scramble shRNA control respectively, the mRNA level of NEDD4-1 increased significantly after NEDD4-1 overexpression but decreased by 70~80% after shNEDD4-1 transfection, indicating that the gene manipulation were successful in vitro. Furthermore, the above results were further confirmed by western blotting assay (Figure 1B). 

Then, the effect of NEDD4-1 on cell migration was determined by wound-healing assay. As Figure 1C and 1D showed, 24h after being scratched, the wound of NEDD4-1 overexpressing group healed obviously and had the tendency to fuse, but the vector control group had no signs of healing. Compared with the control group, the migratory cell number of NEDD4-1 overexpressing group increased by 61% (*P<0.05, Figure 1C and 1D). On the contrary, the migratory cell number of shNEDD4-1 transfection group decreased by 50% compared with the scramble siRNA transfection cells (*P<0.05, Figure 1C and 1D). To rule out the disturbing effect of non-transfected cells, we co-transfected GFP (as an index of transfection efficiency) with NEDD4-1 (a non-GFP vector) at a ratio of 1:3 and took the pictures of GFP positive cells after being scratched for 24h. We found that the GFP transfection efficiency was as high as 65% and the number of migratory GFP positive cells in NEDD4-1 overexpressing group increased by 63% (*P<0.01, Figure S1A). In addition, the effect of shNEDD4-1 on glioma cell migration was also determined by GFP fluorescence and the result was similar to that of bright field (*P<0.01, Figure S1A). We also performed the same experiments in U87 cells and got the similar results (*P<0.01, Figure S1B). The above results indicate that the effect of NEDD4-1 on glioma cell migration was really induced by NEDD4-1 manipulation and was not caused by non-transfection cells. Furthermore, to rule out the potential non-specific (off-target) effects of the shNEDD4-1, we did the rescue experiments by co-transfecting a large amount of NEDD4-1 with shNEDD4-1. We found that shNEDD4-1 cannot down-regulate the large amount of exogenous NEDD4-1 anymore and the exogenous NEDD4-1 can abolish the inhibition effect of shNEDD4-1 on glioma cell migration both in U251 and U87, suggesting that the effect of shNEDD4-1 on cell migration was specific (*P<0.01, Figure S1A and S1B).

As migration and invasion have close inter-communication and NEDD4-1 manipulation affected the migration of human glioma cells, we next examined the role of NEDD4-1 in invasion of glioma cells using matrigel precoated transwell chambers. We found that, after transfection for 48h, overexpression of NEDD4-1 promoted the cell invasion while downregulation inhibited it (Figure 1E and F). Furthermore, the effect of NEDD4-1 on glioma cell migration and invasion was also observed in U87 glioma cells through either wound-healing assay or transwell invasion assay (Figure 1G-1I). To examine whether the migration effect of NEDD4-1 was the secondary effect of proliferation, we also checked the effect of NEDD4-1 on glioma cell proliferation and apoptosis. As Figure S2A showed, neither NEDD4-1 overexpression nor downregulation affected the proliferation of U251 and U87 glioma cells. The cell apoptosis rate also did not show any change after either NEDD4-1 overexpression or downregulation (Figure S2B). These results demonstrate that NEDD4-1 is directly involved in promoting cell migration and invasion. Importantly, this result reminds us that the molecules responsive to glioma cell invasion might be regulated by NEDD4-1.

### NEDD4-1 ubiquitinates CNrasGEF in glioma cells

NEDD4-1 is a HECT-domain ubiquitin ligase and has multiple substrates [16,24]. Recently, it is reported that NEDD4-1 is a potential proto-oncogene that negatively regulates the tumor suppressor PTEN via ubiquitination [33]. However, U251 and U87 glioma cells has mutated PTEN [41,42] and our results showed that either overexpression or downregulation of NEDD4-1 could affect the migration and invasion of U251 and U87 glioma cells (Figure 1), suggesting that these effect of NEDD4-1 might not be mediated by PTEN. 

It is reported that CNrasGEF, a guanine nucleotide exchange factor for small G protein Rap1 [43], is a substrate of NEDD4-1 because CNrasGEF possesses two PY motifs at its C-terminus that are responsible for binding to NEDD4-1 [24,44]. In addition, overexpression of CNrasGEF promotes cell apoptosis, while knocking-down of CNrasGEF inhibits it in melanoma [45]. Furthermore, CNrasGEF substrate Rap1 activation inhibits the cell migration and enhances cell adhesion through regulating E-cadherin [46-48]. Thus, we deduce that the effect of NEDD4-1 on glioma cell migration and invasion might be mediated by CNrasGEF. 

**Figure 2:**
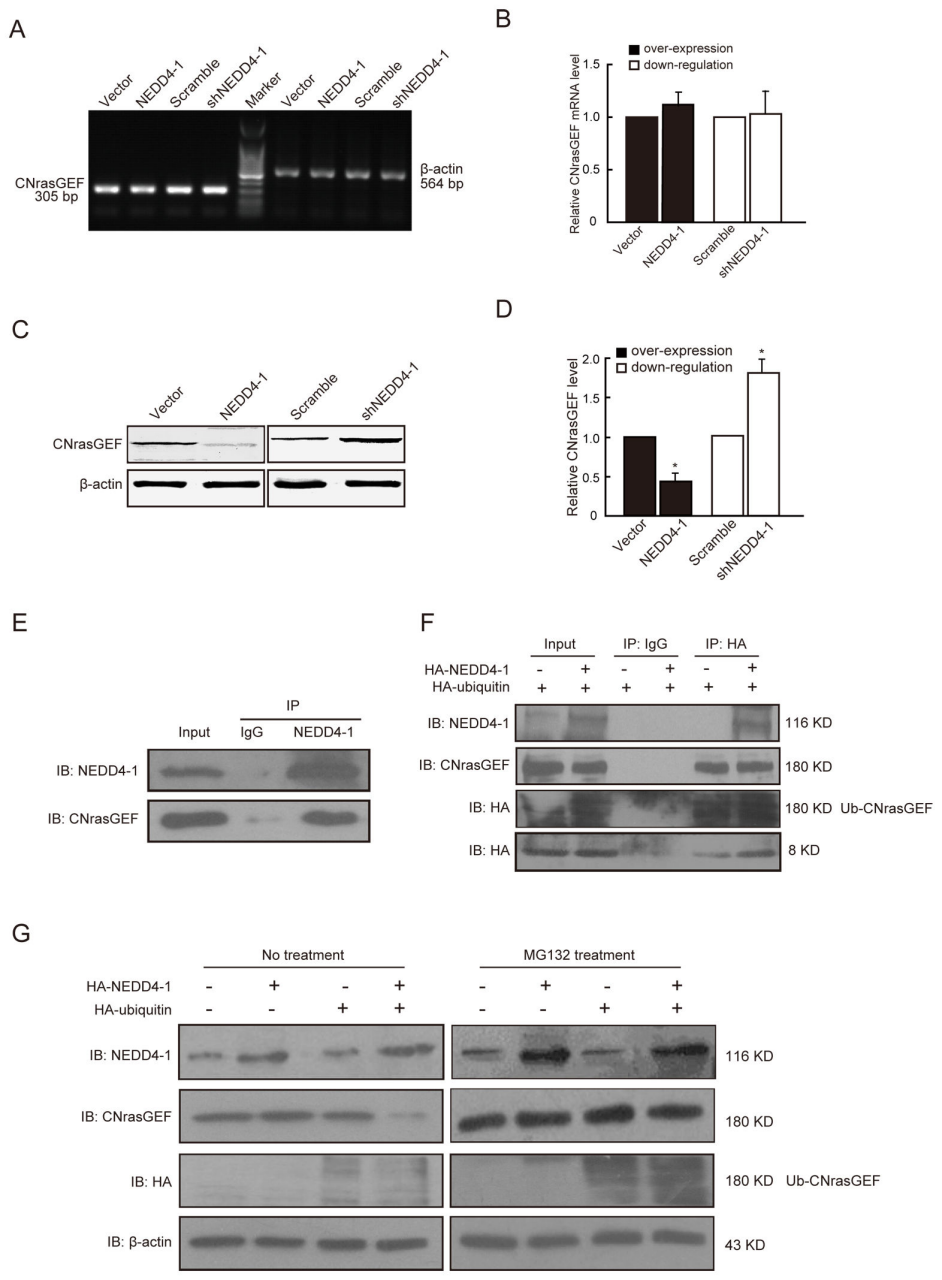
NEDD4-1 ubiquitinates CNrasGEF in glioma cells. A) Representative RT-PCR analysis showed that NEDD4-1 overexpression or downregulation has no effect on CNrasGEF mRNA level. Twenty-four hours after transfection, total RNAs were extracted and underwent RT-PCR analysis. β-actin was used as internal control. B) Quantitative results of A). C) Western blot showed that NEDD4-1 overexpression or downregulation regulated the protein level of CNrasGEF. Forty-eight hours after transfection, cells were lysed and protein extraction was underwent western blot analysis using CNrasGEF antibody. β-actin was used as the loading control. D) Quantitative results of C). E) Interaction of CNrasGEF with NEDD4-1. Glioma U251 cells were transfected with pcDNA3.1-NEDD4-1. Lysate from the transfected cells were immunoprecipitated with anti-Nedd4-1 antibodies or anti-IgG to precipitate CNrasGEF and immunoblotted with anti-CNrasGEF antibodies to detect CNrasGEF. F) NEDD4-1 poly-ubiquitinates CNrasGEF. Glioma U251 cells were transfected with pcDNA3.1-NEDD4-1 and HA-ubiquitin. Lysate from the transfected cells were immunoprecipitated with anti-HA or anti-IgG to precipitate CNrasGEF and immunoblotted with anti-CNrasGEF or anti-HA antibodies to detect CNrasGEF. G) Glioma U251 cells were transfected with pcDNA3.1-NEDD4-1 and HA-ubiquitin with or without 26S-proteasome inhibitor MG132 treatment. Western blot showed that the level of poly-ubiquitinated CNrasGEF increased after MG132 treatment. Data were derived from three independent experiments.

Firstly, we examined whether the level of CNrasGEF was regulated by NEDD4-1. As is shown in Figure 2A and 2B, neither overexpression nor downregulation of NEDD4-1 affected the mRNA level of CNrasGEF. On the contrary, the protein level of CNrasGEF decreased after NEDD4-1 overexpression but increased after NEDD4-1 downregulation (Figure 2C and 2D). These results show that NEDD4-1 indeed regulated the protein level of CNrasGEF and this regulation effect happened at the post-translational stage, suggesting the potential ubiquitinating relationship.

Next, we examined whether CNrasGEF interacts with NEDD4-1 in glioma cells using co-immunoprecipitation assays. As is shown in Figure 2E, the exogenous NEDD4-1 was able to co-precipitate with endogenous CNrasGEF. By contrast, control antibody (i.e., IgG) caused no positive bands (Figure 2E), suggesting the specificity of the interaction. To test whether the interaction between NEDD4-1 and CNrasGEF promoted ubiquitination of this exchange factor, we performed an ubiquitination assay. U251 cells were transfected with HA-ubiquitin, in the presence or absence of NEDD4-1 (HA-NEDD4-1) overexpression, and/or combinations of control vectors. CNrasGEF was then immunoprecipitated from transfected U251 cells using anti-HA antibody and subsequently immunoblotted with anti-HA to detect ubiquitinated CNrasGEF. As is shown in Figure 2F, the endogenous CNrasGEF was ubiquitinated by exogenous HA-ubiquitin, with a characteristic ladder indicative of poly-ubiquitination. In the presence of exogenous NEDD4-1, CNrasGEF protein was heavily ubiquitinated. By contrast, control antibody (i.e., IgG) caused no positive bands (Figure 2F), suggesting the specificity of this effect. These results demonstrate that ubiquitination of CNrasGEF required binding to NEDD4-1, further confirming that NEDD4-1 is the E3 responsible for the ubiquitination of CNrasGEF in glioma U251 cells. Consistently, we also found that the poly-ubiquitinated CNrasGEF was the substrate for proteasomes, because treatment of cells with the proteasome inhibitor MG132 caused a robust increase of CNrasGEF polyubiquitination (Figure 2G). 

### Effects of CNrasGEF on cell migration and invasion

**Figure 3:**
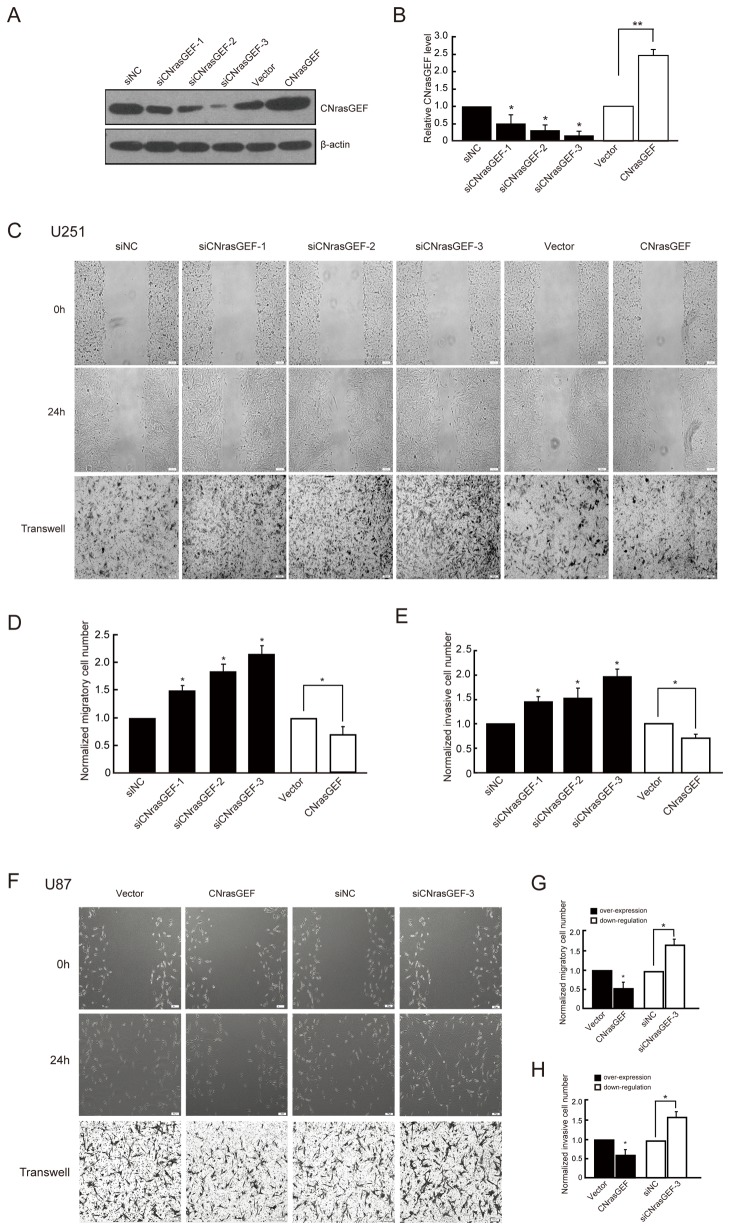
The effect of CNrasGEF on cell migration and invasion. A) Representative western blotting analysis of CNrasGEF knocking-down or overexpression efficacy in U251 glioma cells. B) Quantitative analysis of relative protein levels of CNrasGEF normalized to those of β-actin. C) Wound-healing assay (up and middle panel) and transwell invasion assay (bottom panel) of glioma U251 cells transfected with siRNAs or overexpression of CNrasGEF. D & E) Quantitative analysis of the number of migratory (D) or invasive (E) cells. F) Wound-healing (up and middle panel) and transwell invasion assay (bottom panel) of glioma U87 cells after CNrasGEF downregulation or overexpression. G & H) Quantitative analysis of the number of migratory (G) or invasive (H) cells. Data were mean ± SEM of three independent experiments in triplicate. * *P*<0.05.Bar: 100 μm.

Our results above demonstrated that NEDD4-1 promoted the migration and invasion of the glioma U251 and U87 cells and CNrasGEF ubiquitination and degradation. Then, we tested whether CNrasGEF also has a role in glioma cell migration. We used the RNA interference approach to down-regulate CNrasGEF expression and to observe the effect of CNrasGEF on cell migration and invasion. Three sets of siRNA duplexes (siCNrasGEF-1, siCNrasGEF-2, siCNrasGEF-3) targeted to human CNrasGEF were screened for their efficacy on suppressing CNrasGEF expression and nontargeting siRNA (siNC) was used as a control. We found that all three sets of siRNA duplexes effectively decreased CNrasGEF level by 50~80% (*P<0.05, Figure 3A and 3B). 

Next, we examined whether downregulation of CNrasGEF could impact on migration of glioma cells using wound healing assay. As Figure 3C showed, 24h after being scratched, the wound of CNrasGEF downregulation groups healed obviously and had the tendency to fuse, but the siNC group had no signs of healing. Compared with the control group, the migratory cell numbers of CNrasGEF downregulation groups increased by 63% (**P<0.01), 117% (**P<0.01) and 132% (**P<0.01) respectively (Figure 3C and 3D). Similarly, we also found that downregulation of CNrasGEF also promoted the cell invasion behavior using transwell invasion assay (Figure 3C and 3E). On the contrary, overexpression of CNrasGEF inhibited the cell migration and invasion (Figure 3A-3E). Furthermore, we also found the similar effects of CNrasGEF on cell migration and invasion in U87 glioma cells (Figure 3F-3H). 

### CNrasGEF mediates the effect of NEDD4-1 on cell migration and invasion

**Figure 4:**
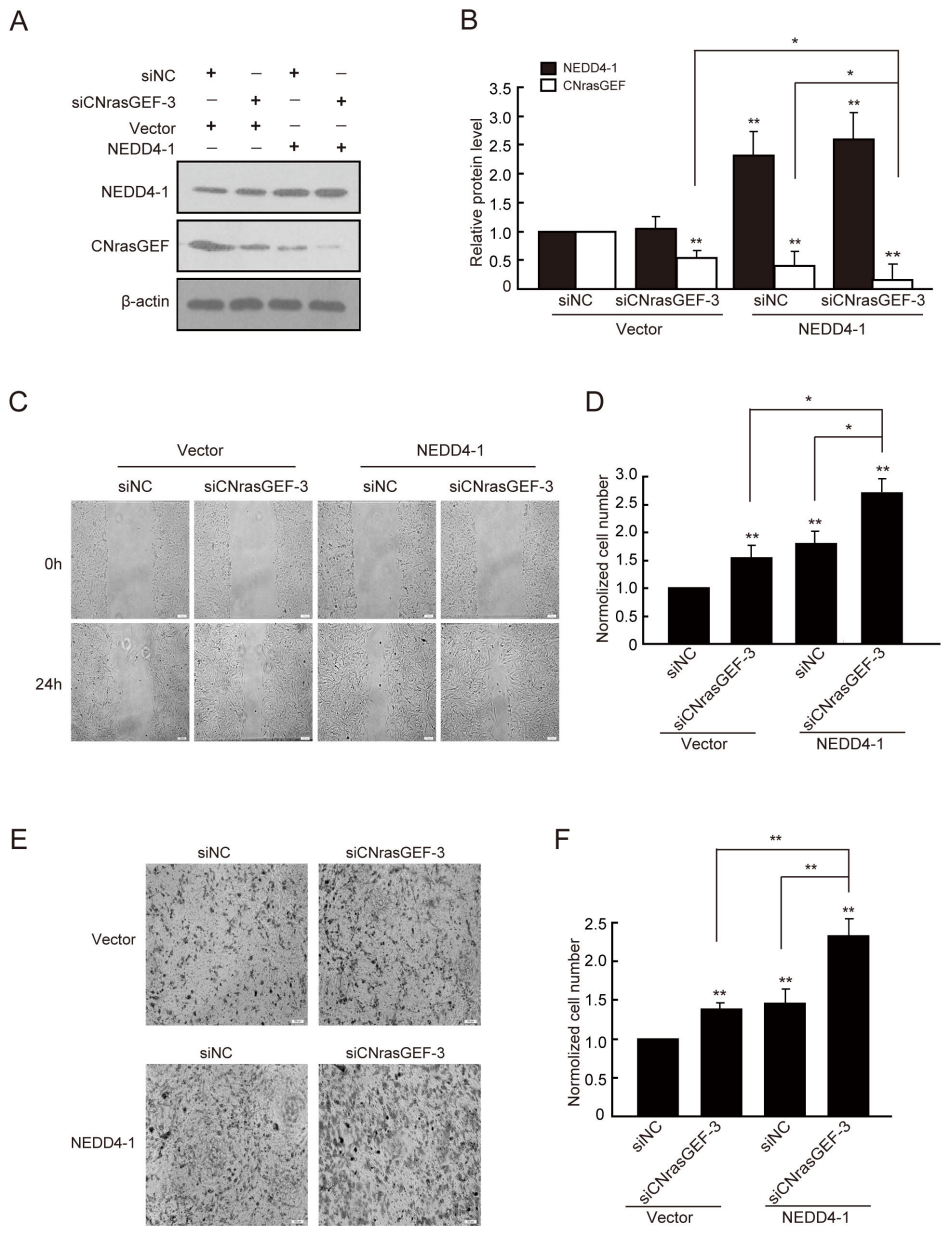
CNrasGEF downregulation facilitates the promotion effect of NEDD4-1 on cell migration and invasion. A) Western blotting showed the expression of NEDD4-1 and CNrasGEF after co-transfection in U251 cells. Representative western blotting analysis of total cell lysates isolated from U251 cells co-transfected with NEDD4-1 and siCNrasGEF using indicated antibodies. B) Quantitative analysis of relative protein levels of NEDD4-1 and CNrasGEF normalized to those of β-actin. C) Wound-healing assays of glioma U251 cells after co-transfection of NEDD4-1 and siCNrasGEF. Representative digital pictures were taken at 0h and 24h. D) Quantitative analysis of the number of migratory cells. E) Transwell invasion assays of glioma U251 cells after co-transfection of NEDD4-1 and siCNrasGEF. F) Quantitative analysis of the number of invasive cells. Results are mean ± SEM of three independent experiments in triplicate. *P<0.05, ** *P*<0.01. Scale bar: 100μm.

To examine whether CNrasGEF mediated the effect of NEDD4-1 on cell migration and invasion, we co-tansfected NEDD4-1 and siCNrasGEF and tested the migration and invasion behavior of U251 glioma cells. Since siCNrasGEF-3 has the highest downregulation efficacy of the three siRNA duplexes (Figure 3A and 3B), we used it to down-regulate CNrasGEF expression in this experiment. As is shown in Figure 4A and 4B, the suppression efficiency of siCNrasGEF and overexpression of NEDD4-1 were confirmed using western blotting assay (*P<0.05, **P<0.01). In line with the previous result (Figure 2C and 2D), overexpression of NEDD4-1 reduced the level of CNrasGEF significantly (Figure 4A and 4B). Forty-eight hours after transfection and 24h after wounding, either CNrasGEF downregulation or NEDD4-1 overexpression enhanced cell migration significantly (Figure 4C and 4D). Importantly, downregulation of CNrasGEF facilitated the promotion effect of NEDD4-1 overexpression on cell migration(*P<0.05, **P<0.01, Figure 4C and 4D). In addition, similar results were also observed in transwell invasion assay (Figure 4E and 4F). 

**Figure 5:**
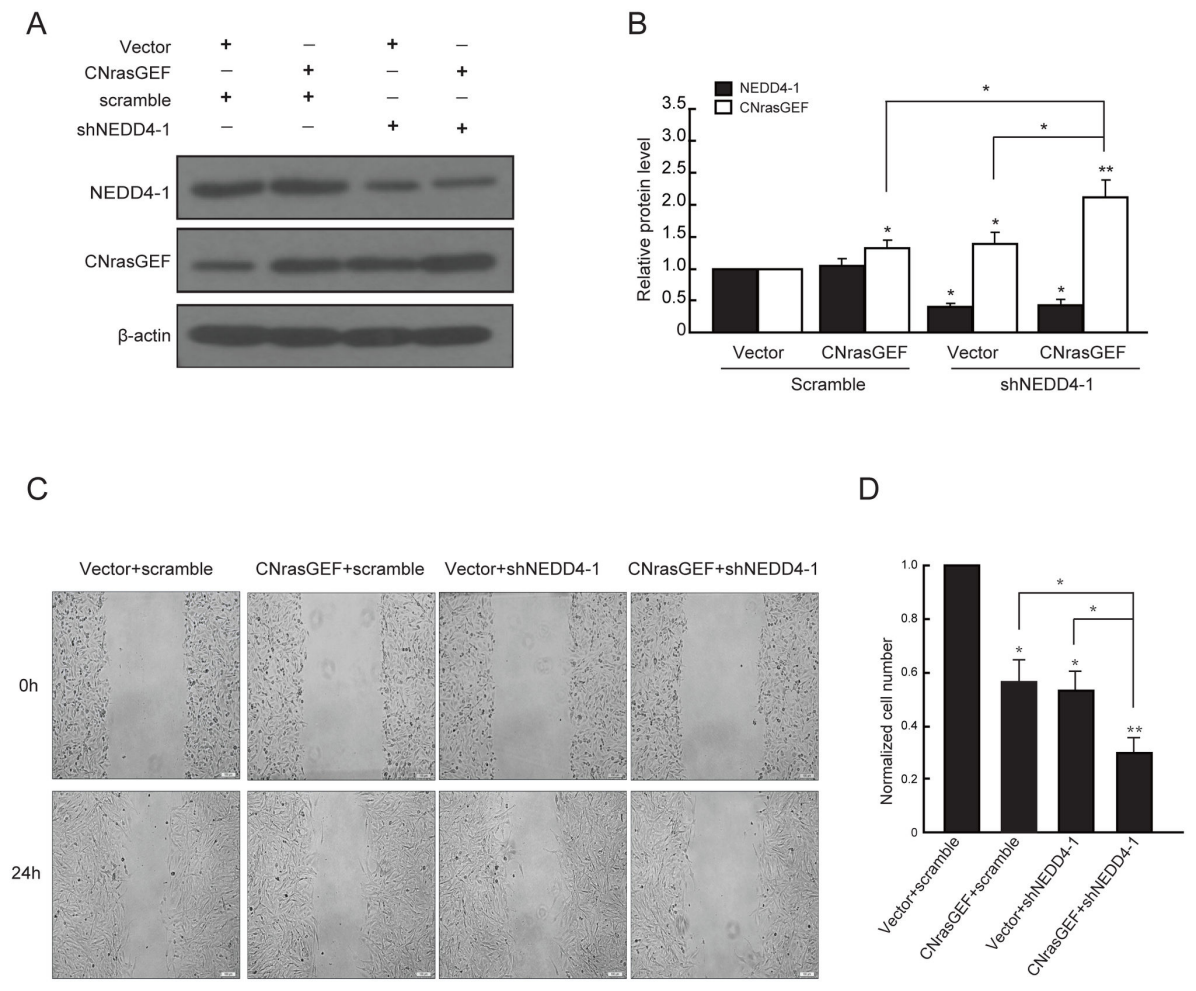
CNrasGEF enhances the inhibition effect of NEDD4-1 downregulation on cell migration and invasion. A) Western blotting showed the expression of NEDD4-1 and CNrasGEF after co-transfection in U251 cells. Representative western blotting analysis of total cell lysates isolated from U251 cells co-transfected with shNEDD4-1 and CNrasGEF using indicated antibodies. B) Quantitative analysis of relative protein levels of NEDD4-1 and CNrasGEF normalized to those of β-actin. C) Wound-healing assays of glioma U251 cells after co-transfection of shNEDD4-1 and CNrasGEF. Representative digital pictures were taken at 0h and 24h. D) Quantitative analysis of the number of migratory cells. Results are mean ± SEM of three independent experiments in triplicate. *P<0.05, ** *P*<0.01. Scale bar: 100μm.

Furthermore, we examined whether co-transfection of shNEDD4-1 and CNrasGEF have the contrary results. As is shown in Figure 5A and 5B, the suppression efficiency of shNEDD4-1 and overexpression of CNrasGEF were confirmed using western blotting assay (*P<0.05, **P<0.01). Forty-eight hours after transfection and 24h after wounding, either NEDD4-1 downregulation or CNrasGEF overexpression reduced cell migration significantly (Figure 5C and 5D). Interestingly, overexpression of CNrasGEF strengthened the inhibition effect of NEDD4-1 downregulation on cell migration (*P<0.05, **P<0.01, Figure 5C and 5D). The above results indicate that the CNrasGEF mediates the effect of NEDD4-1 on cell migration and invasion. 

### Expression of NEDD4-1 and CNrasGEF in human glioma tissues

**Figure 6:**
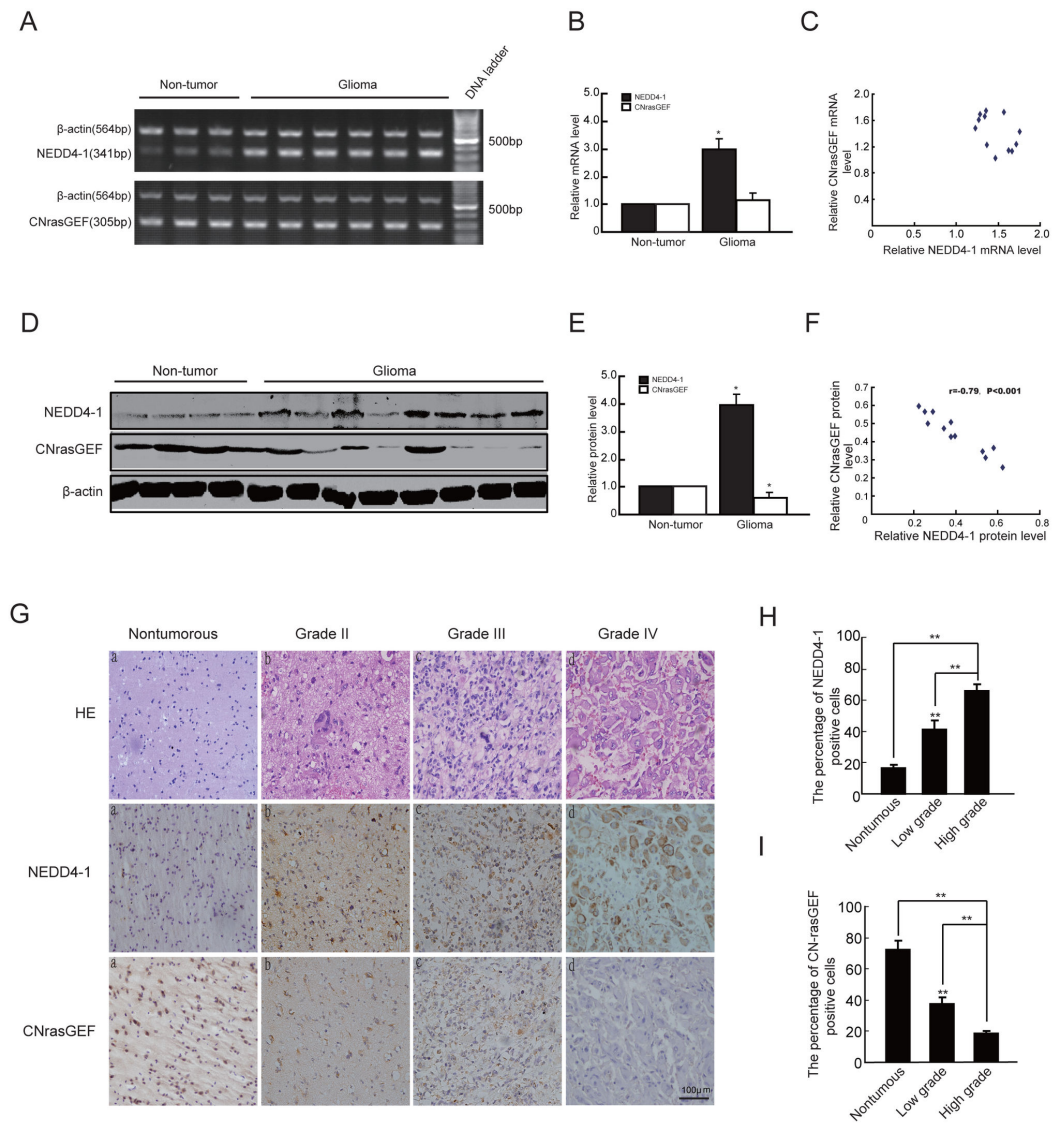
Expression of NEDD4-1 and CNrasGEF in glioma tissues. A) mRNA levels of NEDD4-1 and CNrasGEF in glioma and nontumorous brainspecimens was detected by RT-PCR. B) Quantitative analysis of the mRNA levels of NEDD4-1 and CNrasGEF normalized to those of β-actin. C) The mRNA relationship between the NEDD4-1 and CNrasGEF. D) Western blotting analysis of the total extracts isolated from human nontumorous brain and human glioma specimens using human NEDD4-1 and CNrasGEF antibodies. E) Quantitative analysis of the protein levels of NEDD4-1 and CNrasGEF normalized to those of β-actin. F) The protein relationship between the NEDD4-1 and CNrasGEF. r=-0.79. I) Representative hematoxylin-eosin staining and immunohistochemistry (IHC) analysis of NEDD4-1 and CNrasGEF expression in nontumorous brain tissue and glioma specimens of varying World Health Organization (WHO) grades. Normal brain tissue (a); diffuse astrocytoma, grade II (b); anaplastic astrocytoma, grade III (c); GBM, grade IV (d) are shown. IHC stainings were performed at least twice with similar stained patterns. Scale bar:100μm. J&K) Quantitativeanalysis of the percentage of NEDD4-1 and CNrasGEF positive cells. Data were derived from three independent experiments. ** *P*<0.01 vs nontumorous.

Finally, we studied the clinical relevance of NEDD4-1 and CNrasGEF, and their relationship in clinical glioma samples. In light of the function of NEDD4-1 as CNrasGEF ubiquitin ligase, we hypothesize that overexpression of NEDD4-1 might contribute to the low CNrasGEF protein in these samples. Therefore, we examined the mRNA and protein levels of NEDD4-1 and CNrasGEF in glioma tissues by RT-PCR and western blotting. Representative RT-PCR and western blotting were shown in Figure 6A and 6B. Analysis of the band densities revealed that in 41% of the glioma samples, the mRNA level of NEDD4-1 was approximately 2.8 fold higher than that of nontumorous tissues, but these samples had relatively normal CNrasGEF mRNA levels (Figure 6A and 6B). Thus, NEDD4-1 and CNrasGEF expression showed no correlation at mRNA levels (Figure 6C). However, the result of western blotting showed that the protein levels of NEDD4-1 in 76% of glioma tissues were about 2.1 fold higher than those of nontumorous tissues. Interestingly, the protein levels of CNrasGEF in these glioma tissues were lower than those of nontumorous tissues (Figure 6 B) and there was an inverse correlation between NEDD4-1 and CNrasGEF protein levels (r= -0.79, Figure 6D-6F). 

Furthermore, we assessed the expression and distribution of NEDD4-1 and CNrasGEF in nontumorous and glioma tissues by immunohistochemistry. As is shown in Figure 6G-6I, NEDD4-1 immunoreactivity was located in cytoplasm and nuclei and low (0-+) NEDD4-1 staining scores only occupied 12.46% in normal brain tissues. On the contrary, higher immunoactivity of NEDD4-1 was detected in human gliomas. The NEDD4-1 staining scores of glioma samples were 10.35% to low (0-+), 31.03% (++) moderate and 58.62% strong positive (+++) (*P*<0.01, Figure 6G-6I). In addition, the expression level of NEDD4-1 protein significantly correlated with the malignancy grade of the glioma. As is shown in Figure 6G, the level of NEDD4-1 was significantly higher in low grade gliomas than in normal brain tissues. The higher the grade of gliomas, the higher the level of NEDD4-1 expression (nontumorous=16.15±1.50%, low grade=40.32 ±4.01%, high grade=66.70± 5.44%, *P*<0.01, respectively, Figure 6G-6I).

CNrasGEF was also located in cytoplasm and nuclei, but contrary to that of NEDD4-1, the number of CNrasGEF positive cells in glioma samples was significantly smaller than that in nontumorous brain tissues (*P*<0.01, Figure 6G-6I). The CNrasGEF staining scores of glioma samples were 31.03% moderate (++), and 68.97% negligible to low (0-+). In addition, the higher the grade of gliomas, the lower the level of CNrasGEF expression (nontumorous=72.01±6.36%, lowg rade=37.04±3.88%, high grade=18.03±1.5%, *P*<0.01, respectively, Figure 6G-6I). Collectively, these results further confirmed the inverse correlation between NEDD4-1 and CNrasGEF protein levels. 

## Discussion

In this study, we show that NEDD4-1 plays a key regulatory effect in the migratory and aggressive behavior of glioma via regulating ubiquitination of CNrasGEF. Several lines of evidence conveyed this point. First, overexpression of NEDD4-1 promoted cell migration and invasion, while NEDD4-1 downregulation inhibited them. Second, NEDD4-1 polyubiquitinated CNrasGEF and thus target CNrasGEF for proteasomal degradation. Third, CNrasGEF downregulation promoted the migration and invasion of human glioma cells, CNrasGEF overexpression inhibited them, which is contrary to the effect of NEDD4-1. Interestingly, overexpression of NEDD4-1 and downregulation of CNrasGEF at the same time significantly promoted the migration and invasion, but downregulation of NEDD4-1 and overexpression of CNrasGEF at the same time showed the opposite effect. Fourth, aberrant up-regulated NEDD4-1 showed reverse correlation with CNrasGEF protein level but not with its mRNA level in glioma tissues. 

As a member of NEDD4 family, NEDD4-1 belongs to HECT ubiquitin ligase and regulates the following physiological functions through ubiquitinating various substrates: cellular proliferation and organism growth [32], water balance, T cell function [26] and the development of neuromuscular junction [27], development of central nervous system and axon guidance [16]. Recently, it is reported that NEDD4-1 is highly expressed in a wide variety of tumors, such as colorectal cancer, bladder cancer, gastric carcinoma and involved in cancer cell growth [33-46]. We found that NEDD4-1 was highly expressed both at mRNA and protein levels in human glioma tissues (Figure 6), suggesting that NEDD4-1 might play some roles in glioma progression. Although NEDD4-1 has been reported to promote proliferation of some cancer cells, there is no report about the role of NEDD4-1 in cell migration and aggressive behavior, one of important features of glioma. In this work, we found that NEDD4-1 promotes the glioma cell migration and invasion, suggesting that NEDD4-1 exerts a regulatory effect upon the migration and aggressive behavior of glioma.

NEDD4-1 possesses a C2 domain, 2-4 WW domains, and a HECT-type ligase domain. NEDD4-1 binds the special substrates through its WW domain, while the structure of the substrate typically contains PY domain or LPSY sequence [25]. CNrasGEF is identified as a substrate of NEDD4-1 in a screen for NEDD4-1 WW domain interacting proteins [24,44]. In our study, we found that CNrasGEF was able to interact with NEDD4-1 and was polyubipuitinated by NEDD4-1 and subsequently was degraded in proteasome (Figure 2 E - G) in vitro, which is consistent with the above report. Furthermore, contrary to the expression of NEDD4-1 in glioma tissues, although the mRNA level showed no difference between the nontumorous and glioma tissues, the protein level of CNrasGEF decreased in human glioma tissues (Figure 6A- D). Strikingly, in many human glioma samples where the genetic background of CNrasGEF was normal but its protein levels were low, NEDD4-1 was highly expressed, suggesting that aberrant up-regulation of NEDD4-1 might post-translationally modify CNrasGEF in cancers. Therefore, NEDD4-1 is a potential proto-oncogene that negatively regulates CNrasGEF via ubiquitination.

Studies strongly suggest that CNrasGEF is involved in cancer biology. For example, it is proved that CNrasGEF regulates melanogenesis and cell survival in melanoma cells [45]. We found that CNrasGEF also affects cell migration and invasion, opposite to the effect of NEDD4-1 (Figure 1 C- I; 3 C- H). Interestingly, downregulation of CNrasGEF enhanced the effect of NEDD4-1 overexpression on cell migration and invasion (Figure 4 C- F), while overexpression of CNrasGEF and downregulation of NEDD4-1 at the same time further inhibited cell migration and invasion behavior (Figure 5 C- F). These results indicate that CNrasGEF mediates the effect of NEDD4-1 on cell migration and invasion. Since CNrasGEF substrate Rap1 activation inhibits the cell migration and enhances cell adhesion through regulating E-cadherin [47-49], many studies have also pointed out a strong interaction between invasiveness of human gliomas and degradation of the extracellular matrix by matrix metalloproteases (MMPs). Thus, it will be interesting to elucidate the mechanism of NEDD4-1-CNrasGEF signaling on cell migration and invasion by checking the change of these molecules. 

In summary, we found that NEDD4-1regulates the migration and invasion of glioma cells by mediating the ubiqutination of CNrasGEF in vitro. This finding opens a new avenue to study the roles of CNrasGEF in tumorigenesis and other important processes. It is thus possible that NEDD4-1-CNrasGEFsignaling might be a novel potential therapeutic target for human glioma.

## Supporting Information

Figure S1 **Downregulation of NEDD4-1 induced glioma cell migration decrease is abolished by large amount of exogenous NEDD4-1.** A) Downregulation of NEDD4-1 induced glioma cell migration decrease was rescued by a large amount of NEDD4-1 overexpression in U251 glioma cells. Representative digital pictures were taken at 0h and 24h. Bar: 100 μm (left).Quantitative analysis of the number of migratory GFP positive cells. (*P< 0.05; **P< 0.01. right). B) Downregulation of NEDD4-1 induced glioma cell migration decrease was rescued by high NEDD4-1 overexpression in U87 glioma cells. Representative digital pictures were taken at 0h and 24h. Bar: 100 μm (left). Quantitative analysis of the number of migratory GFP positive cells (*P< 0.05; **P< 0.01. right).
(TIF)

Figure S2 **NEDD4-1 does not affect U251 and U87 glioma cell proliferation and apoptosis.** A) The effect of NEDD4-1 overexpression or downregulation on human U251 and U87 cell proliferation measured by the CCK-8 assay 24h after transfection. B) The effect of NEDD4-1 overexpression or downregulation on human U251 cell apoptosis measured by Vybrant Apoptosis Assay Kit #2 combined with flow cytometry analysis. Representative flow cytometry analysis of cell apoptosis co-stained with Annexin V/PI after NEDD4-1 overexpression or downregulation for 48 h in U251 cells. Control cells were transfected with vector or scramble shRNA. The lower right quadrant in the dot plot represents early stage apoptotic cells (left). Quantitative analysis of the percentage of apoptotic cells in the early stage normalized to that of the control group (right). The results are shown as the mean ± SEM of three independent experiments in triplicate.
(TIF)
