# Supplementary figures and images for: NEDD4-1 Regulates Migration and Invasion of Glioma Cells through CNrasGEF Ubiquitination In Vitro
Source: PLoS One. 2013 Dec 10;8(12):e82789. doi: 10.1371/journal.pone.0082789 (PMC3858320; doi:10.1371/journal.pone.0082789)

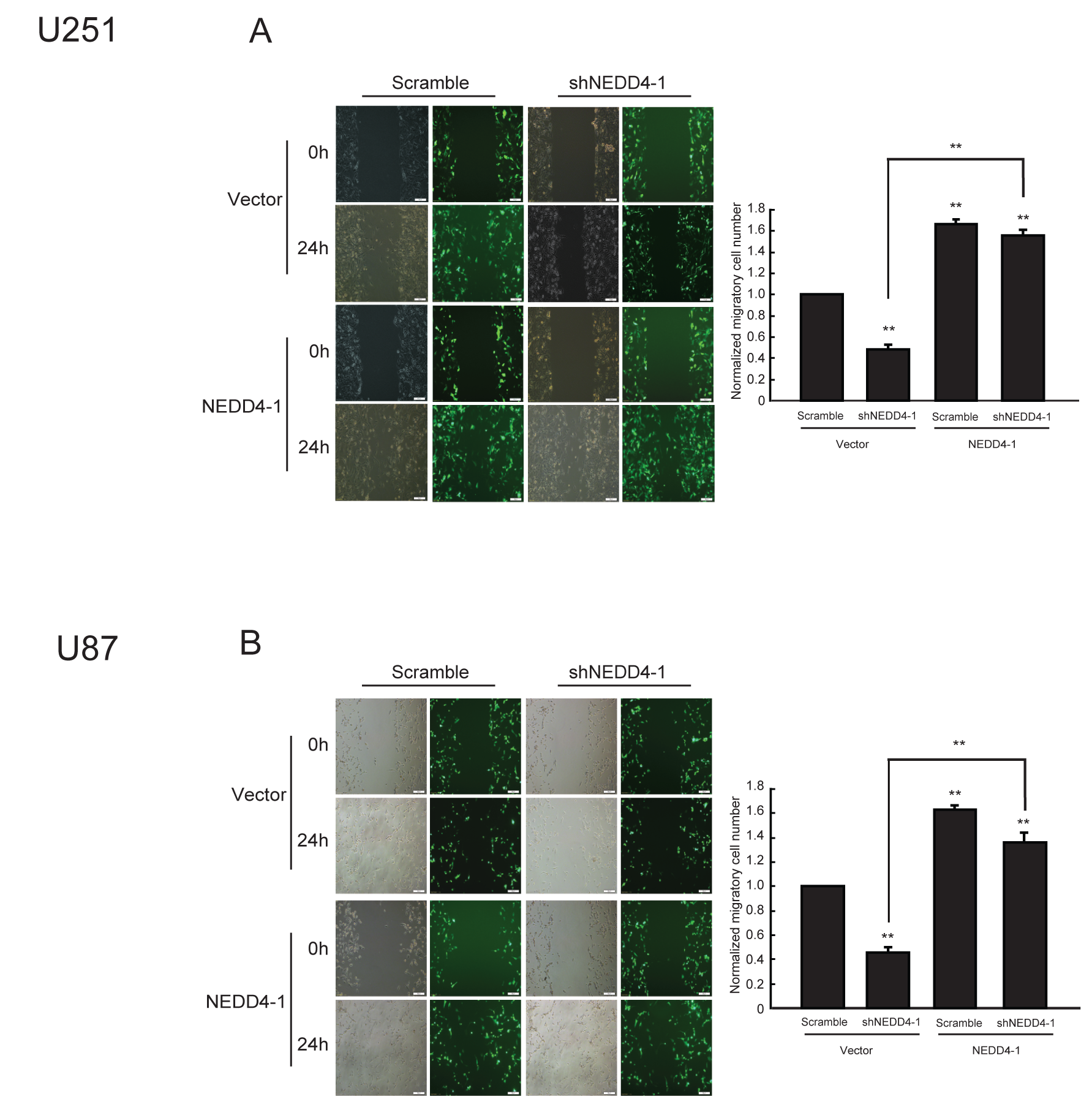

Supplement: Figure S1 — Downregulation of NEDD4-1 induced glioma cell migration decrease is abolished by large amount of exogenous NEDD4-1. A) Downregulation of NEDD4-1 induced glioma cell migration decrease was rescued by a large amount of NEDD4-1 overexpression in U251 glioma cells. Representative digital pictures were taken at 0h and 24h. Bar: 100 μm (left).Quantitative analysis of the number of migratory GFP positive cells. (*P< 0.05; **P< 0.01. right). B) Downregulation of NEDD4-1 induced glioma cell migration decrease was rescued by high NEDD4-1 overexpression in U87 glioma cells. Representative digital pictures were taken at 0h and 24h. Bar: 100 μm (left). Quantitative analysis of the number of migratory GFP positive cells (*P< 0.05; **P< 0.01. right). (TIF) [file pone.0082789.s001.tif]

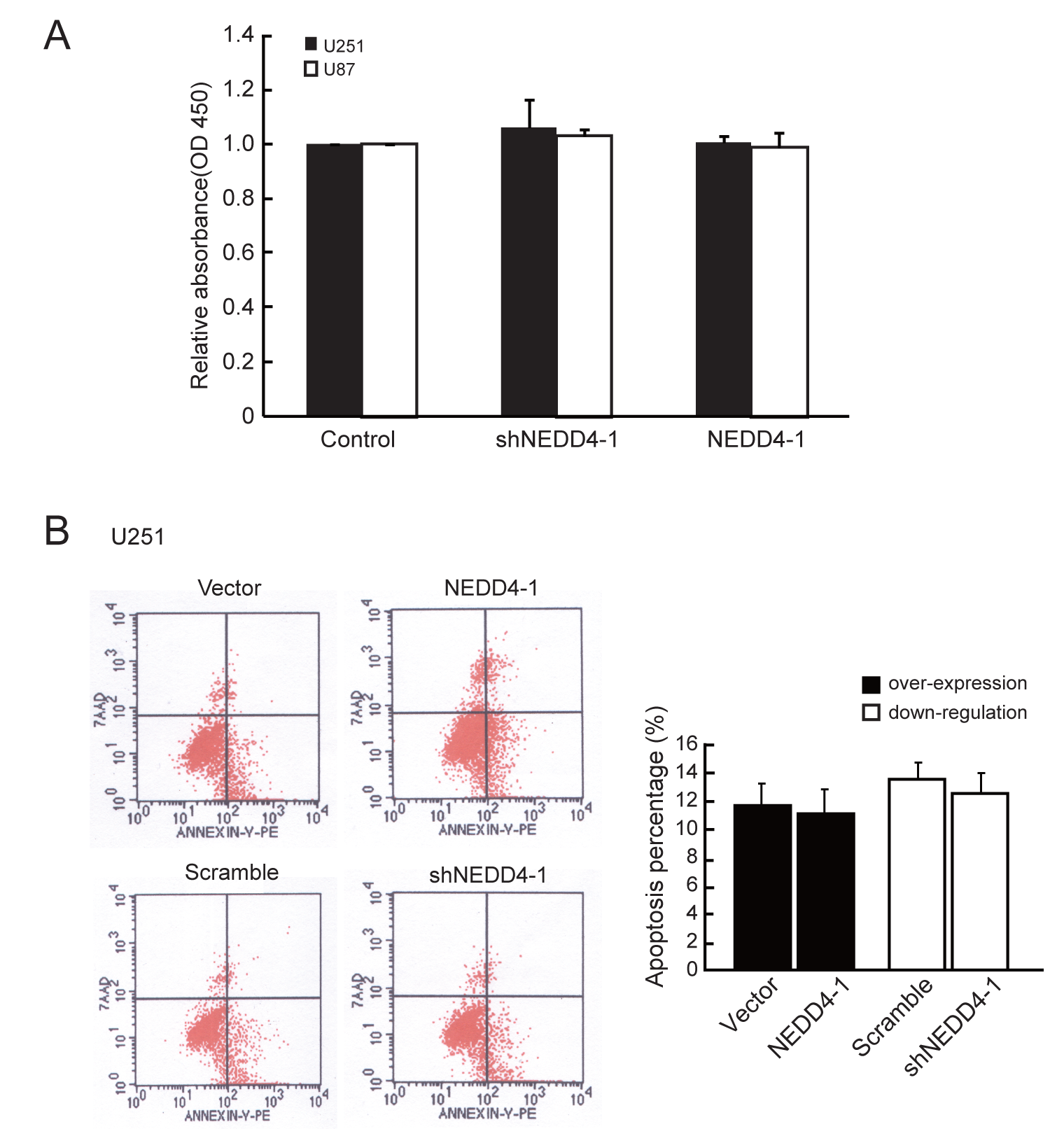

Supplement: Figure S2 — NEDD4-1 does not affect U251 and U87 glioma cell proliferation and apoptosis. A) The effect of NEDD4-1 overexpression or downregulation on human U251 and U87 cell proliferation measured by the CCK-8 assay 24h after transfection. B) The effect of NEDD4-1 overexpression or downregulation on human U251 cell apoptosis measured by Vybrant Apoptosis Assay Kit #2 combined with flow cytometry analysis. Representative flow cytometry analysis of cell apoptosis co-stained with Annexin V/PI after NEDD4-1 overexpression or downregulation for 48 h in U251 cells. Control cells were transfected with vector or scramble shRNA. The lower right quadrant in the dot plot represents early stage apoptotic cells (left). Quantitative analysis of the percentage of apoptotic cells in the early stage normalized to that of the control group (right). The results are shown as the mean ± SEM of three independent experiments in triplicate. (TIF) [file pone.0082789.s002.tif]
